# Degrading an enzyme to increase its product: a novel approach to decoupling biosynthesis and growth

**DOI:** 10.1093/synbio/ysz001

**Published:** 2019-01-10

**Authors:** Ciarán L Kelly

**Affiliations:** Agriculture and Engineering, School of Natural and Environmental Sciences, Newcastle University, Newcastle upon Tyne, UK

In biomanufacturing, it is often desirable to uncouple enzyme levels and activities from bacterial growth rates. When an enzyme-encoding gene that is used to produce a target chemical is constitutively expressed, production of the chemical of choice is limited, as most of the carbon is diverted into biomass production. Many different approaches have been used to tackle this problem. Most commonly, gene expression is turned on at specific phases in bacterial growth to control the timing of protein production. A recent article in *ACS Synthetic Biology* introduces ‘FENIX’, a novel, post-translational system for uncoupling biosynthesis from growth.^1^

While most uncoupling strategies rely on an ‘on switch’ to delay the induction of gene expression, the unique FENIX system is an ‘off switch’ that stops the protein of interest from being degraded. FENIX combines two different protein-degradation machineries, one that is native to the *Escherichia coli* host and one that is introduced. A sequence encoding a C-terminal Ssra peptide tag is fused to the gene of interest, which is expressed from a plasmid. This tag results in continuous degradation of the protein of interest by proteases always present in the host organism (ClpXP and ClpAP). Encoded immediately upstream of the Ssra tag is another peptide tag that is recognized by the non-native protease NIa (used in viral polypeptide processing). NIa is encoded on a second plasmid under the control of an inducible promoter. When expression of the gene encoding NIa is induced, cleavage of the polypeptide occurs at the NIa target site, removing the Ssra tag from the rest of the protein. Because the gene of interest was already being expressed, turning off degradation allows for rapid accumulation of the protein.

The authors initially used fluorescent proteins to successfully demonstrate that the FENIX system enables both tight control and rapid induction of a protein of interest. They then applied the system to the production of the renewable plastic polymer, polyhydroxybutrate (PHB). Many groups have taken the genes encoding the three enzymes required for PHB production, *phaC*, *phaA* and *phaB* from *Cupriavidus necator*, and expressed them in *E. coli*,^2^^–^^5^ with limited yields to date. The substrate for PHB production is acetyl-CoA, one of the main hubs for carbon and electron flow in metabolism. The authors hypothesized that by decoupling PhaA enzyme accumulation and activity from the exponential phase of bacterial growth, competition for acetyl-CoA would be reduced and greater rates and overall yields of PHB production could be achieved. They showed that by using the FENIX system to control the levels of PhaA (and constitutively expressing *phaB* and *phaC*), growth-independent accumulation of PhaA activity and PHB production was possible. Finally, the authors demonstrated that this system could operate as a post-translational metabolic switch, allowing diversion of carbon and electrons away from acetate production towards PHB production.

Overall, the FENIX post-translational system presented in this work, represents an intriguing alternative approach to tackling the challenge of precise temporal control of enzyme levels *in vivo*. It will be interesting to compare the effect of continuous production and degradation of a protein on overall cellular burden with transcriptional and translational regulatory approaches. One exciting potential use of this system could be in the construction of metabolic feedback circuits, a current area of intense activity in synthetic metabolic engineering.

## References

[1] Durante-Rodríguez G., de Lorenzo V., Nikel P.I. (2018) A post-translational metabolic switch enables complete decoupling of bacterial growth from biopolymer production in engineered *Escherichia coli*. ACS Synth. Biol., 7, 2686–2697. 10.1021/acssynbio.8b00345 30346720

[2] Lee S.Y., Lee Y. (2003) Metabolic engineering of *Escherichia coli* for production of enantiomerically pure (R)-(–)-hydroxycarboxylic acids. Appl. Environ. Microbiol., 69, 3421–3426. 12788745 10.1128/AEM.69.6.3421-3426.2003 PMC161469

[3] Schubert P., Steinbüchel A., Schlegel H.G. (1988) Cloning of the Alcaligenes eutrophus genes for synthesis of poly-beta-hydroxybutyric acid (PHB) and synthesis of PHB in *Escherichia coli*. J. Bacteriol., 170, 5837–5847. 2848014 10.1128/jb.170.12.5837-5847.1988 PMC211690

[4] Slater S.C., Voige W.H., Dennis D.E. (1988) Cloning and expression in *Escherichia coli* of the Alcaligenes eutrophus H16 poly-beta-hydroxybutyrate biosynthetic pathway. J. Bacteriol., 170, 4431–4436. 3049530 10.1128/jb.170.10.4431-4436.1988 PMC211473

[5] Saegusa H., Shiraki M., Kanai C., Saito T. (2001) Cloning of an intracellular Poly[D(-)-3-Hydroxybutyrate] depolymerase gene from Ralstonia eutropha H16 and characterization of the gene product. J. Bacteriol., 183, 94–100. 11114905 10.1128/JB.183.1.94-100.2001 PMC94854

